# Electronic Structure and Ferromagnetism Modulation in Cu/Cu_2_O Interface: Impact of Interfacial Cu Vacancy and Its Diffusion

**DOI:** 10.1038/srep15191

**Published:** 2015-10-19

**Authors:** Hao-Bo Li, Weichao Wang, Xinjian Xie, Yahui Cheng, Zhaofu Zhang, Hong Dong, Rongkun Zheng, Wei-Hua Wang, Feng Lu, Hui Liu

**Affiliations:** 1 Department of Electronics and Tianjin Key Laboratory of Photo-Electronic Thin Film Device and Technology, Nankai University, Tianjin, 300071, China; 2 School of Materials Science and Engineering, Hebei University of Technology, Tianjin, 300130, China; 3 School of Physics, the University of Sydney, NSW, 2006, Australia; 4 Department of Materials Science and Engineering, The University of Texas at Dallas, Richardson, Texas 75080, United States

## Abstract

Cu/Cu_2_O composite structures have been discovered to show sizable ferromagnetism (FM) with the potential applications in spintronic devices. To date, there is no consensus on the FM origin in Cu/Cu_2_O systems. Here, first principles calculations are performed on the interface structure to explore the microscopic mechanism of the FM. It is found that only the Cu vacancy (V_Cu_) adjacent to the outermost Cu_2_O layer induces a considerable magnetic moment, mostly contributed by 2*p* orbitals of the nearest-neighbor oxygen atom (O_NN_) with two dangling bonds and 3*d* orbitals of the Cu atoms bonding with the O_NN_. Meanwhile, the charge transfer from Cu to Cu_2_O creates higher density of states at the Fermi level and subsequently leads to the spontaneous FM. Furthermore, the FM could be modulated by the amount of interfacial V_Cu_, governed by the interfacial Cu diffusion with a moderate energy barrier (~1.2 eV). These findings provide insights into the FM mechanism and tuning the FM via interfacial cation diffusion in the Cu/Cu_2_O contact.

Diluted magnetic semiconductors (DMS) are promising materials for spintronic devices due to the employment of the spin degree of freedom besides the charge property of carriers^1^,^2^. Among the III-V semiconductors doped by transition metals, spin polarization of carriers is achieved in Mn-doped GaAs, InAs and etc^3^,^4^. However, the low Curie temperature far below room temperature impedes the DMS-based device development^5^. In recent years, oxides diluted magnetic semiconductors (ODMS), such as Fe-ZnO, Cr-TiO_2_, Mn-TiO_2_ and etc, have attracted much attention due to their Curie temperature over 300 K in regards to DMS^6^,^7^,^8^. Unexpectedly, room temperature ferromagnetism (FM) has been observed in undoped oxides. After Venkatesan and Coey *et al.* pioneered in the finding of the room-temperature FM in undoped HfO_2_^9^, other oxides without intentional magnetic impurities doping are subsequently discovered to show the FM behavior^10^,^11^,^12^.

Significant investigations of the FM mechanism are performed on the undoped nanoscaled ODMS systems, such as fine powder, heterostructures, core-shell nanoparticles and etc, where the surface and interfacial native point defects are supposed to be the origin of the FM^13^,^14^,^15^,^16^. However, without the contribution of magnetic transition metal ions, the traditional magnetic exchange interactions in oxides, including long range double-exchange or super-exchange, are no longer suitable for depicting the FM in these systems. Therefore, a charge transfer Stoner model is proposed to fundamentally understand the FM induced by defects^17^,^18^,^19^. The criterion of Stoner model requires *D*(*E*_F_)*J* > 1, where *D*(*E*_F_) is the density of states at the Fermi level (*E*_F_) and *J* denotes the strength of exchange interaction. Spontaneous FM is triggered when a large *D*(*E*_F_) occurs. Nevertheless, it is still not accessible how surface and interfacial defects introduce the localized states around Fermi level, and finally result in a large *D*(*E*_F_) through the charge transfer. It is thus critical to unveil the connection between the FM and the defects states.

Relative to *n*-type ODMS, the *p*-type ODMS are much less explored. As a natural *p*-type material, cuprous oxide (Cu_2_O) is a promising material as catalyst, transistors, and etc^20^,^21^. If Cu_2_O could possess room-temperature FM, Cu_2_O would not only act as one spintronic material but also offer a fundamental platform to study the correlation between FM and structural properties. Actually, cuprous oxide (Cu_2_O) has shown room-temperature FM as other undoped oxides^9^,^10^,^11^,^12^. To date, there is no consensus of the origin of the FM in undoped Cu_2_O. Theoretically, the FM in undoped Cu_2_O is claimed to be induced by the oxygen interstitial in the bulk and by unsaturated Cu in the surface^22^. Experimentally, the undoped Cu_2_O fine powder^13^, nanowires^23^, and CuO/Cu_2_O interface^24^ all exhibit the room-temperature FM relevant with the cation defects. Especially in our previous experimental work in Cu/Cu_2_O core-shell nanoparticles, the FM is found to be closely connected with the Cu vacancy (V_Cu_)^25^. Besides, the magnetization can be tuned by modulating the V_Cu_ amount through controlling the oxygen partial pressure and the annealing duration. Basically, it is critical to clarify the microscopic mechanism of V_Cu_ induced FM and the feasibility to tune the FM through V_Cu_ generation or compensation.

In this work, we have performed the density functional theory (DFT) calculations on the Cu/Cu_2_O interface in order to elucidate the origin and the modulation of FM. Our results indicate that the defect-free Cu/Cu_2_O interface is nonmagnetic, but in contrast, the interface containing specific site of V_Cu_ possesses FM. The FM is not directly contributed by the V_Cu_ but mostly contributed by the nearest-neighbor oxygen atom (O_NN_) and the Cu atoms (Cu_NN_) bonding with the O_NN_. As the anti-bonding states between the O_NN_ and the Cu_NN_ form localized states near the valence band maximum (VBM), the *E*_F_ is pinned at the localized states and a large *D*(*E*_F_) is satisfied through the charge-transfer from Cu to Cu_2_O at the interface. A moderate energy barrier (~1.2 eV) of the Cu diffusion guarantees the feasibility of modulating the FM through controlling the amount of interfacial V_Cu_. Our results provide an insight to understand the origin and the modulation of the FM in Cu/Cu_2_O contact.

## Results and Discussion

### Stabilities of Cu/Cu_2_O interfaces with different interfacial Cu and O contents

The Cu(111) surface is the most stable in terms of the surface coordination^26^. When the Cu(111) surface is oxidized, an intrinsic Cu(111)/Cu_2_O(111) interface forms^27^,^28^. In this work, we focus on the Cu(111)/Cu_2_O(111) intrinsic interface, labeled as Cu/Cu_2_O. The bulk and surface calculations ensure that five layers slab model is suitable for the further interface exploration (See Supplementary Information).

**Figure 1:**
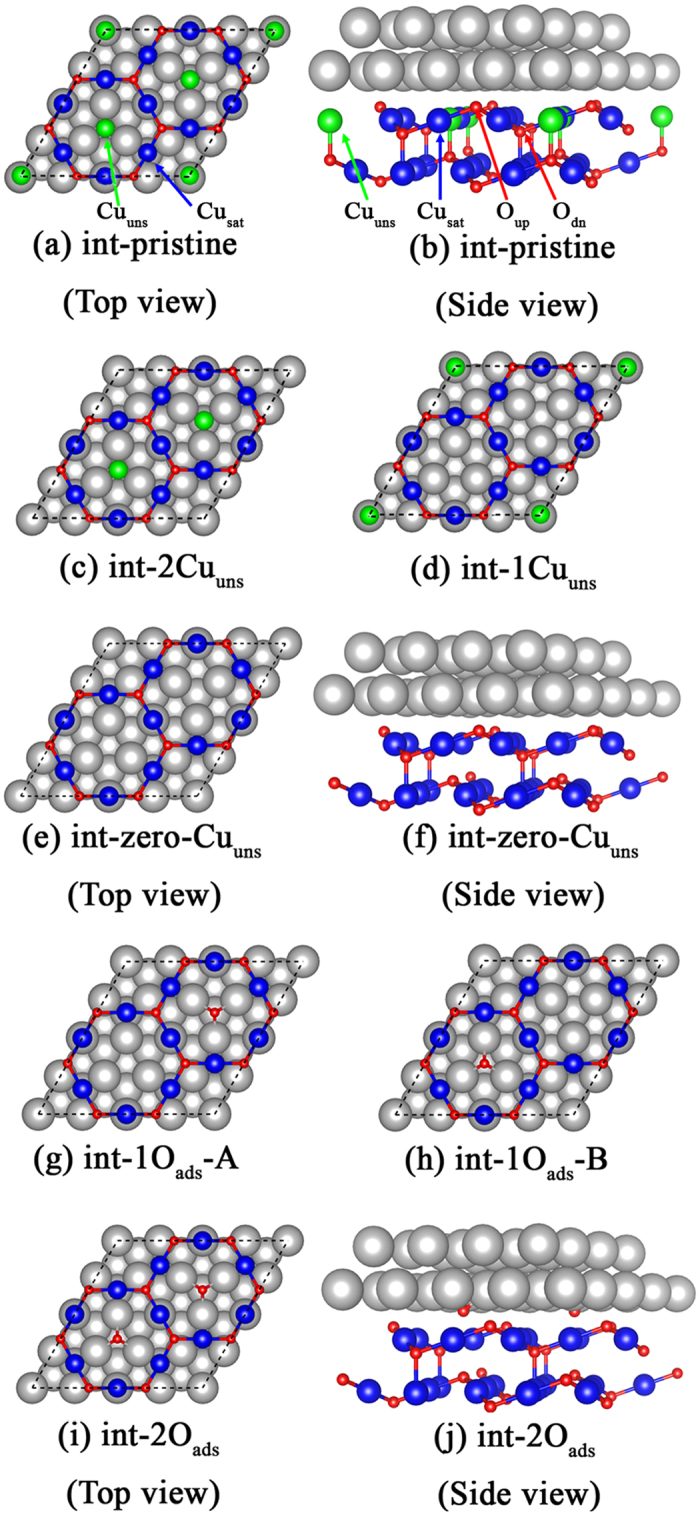
Top and side views of Cu/Cu_2_O interface with pristine structure, int-pristine in (**a**) and (**b**). Part of the interfacial Cu_uns_ atoms present, two and one Cu_uns_ in (**c**) int-2Cu_uns_ and (**d**) int-1Cu_uns_). Top and side views of interface without interfacial Cu_uns,_ int-zero-Cu_uns_ in (**e**,**f**). Interfaces with additional adsorbed oxygen atoms based on the int-zero-Cu_uns_. According to the O_ads_ locations and amount, they are labeled as int-1O_ads_-A in (**g**), int-1O_ads_-B in (**h**) and int-2O_ads_ in (**i**,**j**). Large silver balls are Cu atoms in Cu(111) surface. Green and blue balls denote the Cu_uns_ and Cu_sat_ in Cu_2_O, respectively. Small red balls are oxygen atoms.

Five sandwich-like layers of Cu_2_O(111) surface and five layers of Cu(111) surface are included in a slab model with a 15 Å vaccum space, where the top Layer 1 (see Supplementary Information Figure S1) of the Cu_2_O(111) contacts with the Cu(111) and the bottom Layer 5 is passivated. In practice, oxide surface reconstruction plays an important role to influence the interface quality. Thus, in order to capture this important structural information, we consider Cu_2_O surface reconstruction here. As a recent scanning tunneling microscopy (STM) result proposed, under different oxidation conditions, 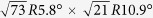 structure (also known as the “44” surface) is one of the stable Cu_2_O(111) surface reconstructions^26^. Therefore, in this work, we concentrate on the interface Cu/Cu_2_O based on this specific “44” surface reconstruction. Referring to Soon *et al.*^29^, we constructed the Cu/Cu_2_O interface by a 2 × 2 Cu(111) surface and a 4 × 4 Cu_2_O(111) surface. Due to the better ductibility of the metal Cu than semiconductor Cu_2_O, the lattice parameters of Cu_2_O are kept constant in Cu/Cu_2_O interface structures. The lattice mismatch for Cu is ~2.7%. According to the interfacial copper and oxygen contents, several candidate interface structures are considered. Figure 1(a,b) are top and side views of the pristine Cu/Cu_2_O interface (int-pristine). Figure 1(c,d) present two and one Cu_uns_ atoms in the Cu_2_O part, respectively (int-2Cu_uns_, int-1Cu_uns_). Figure 1(e,f) are top and side views of the interface without Cu_uns_ atoms in the Cu_2_O part (int-zero-Cu_uns_). Figure 1(g,j) are interfaces with additional adsorbed oxygen atoms based on the int-zero-Cu_uns_. According to the O_ads_ locations and amount, they are labeled as int-1O_ads_-A, int-1O_ads_-B and int-2O_ads_, respectively.

To determine the most stable interface, the interface free (formation) energy is calculated using the following equation,


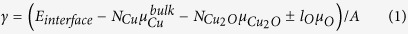


where *E*_interface_ is the interface total energy. *N*_*Cu*_ and 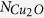 denote the amount of Cu atom and Cu_2_O primitive unit, respectively. 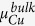, and *μ*_*O*_ are the chemical potentials of bulk Cu, and oxygen in bulk Cu_2_O. Here, 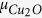 represents the energy of one Cu_2_O primitive cell. *l*_*O*_ is the number of the excess or deficient oxygen atoms in the interface. *A* is the interface area. The chemical potentials 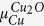 and *μ*_*O*_ in Cu_2_O possess the relationship of:


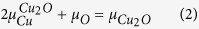


where 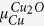 is the chemical potential of Cu in Cu_2_O.

As we know, the maximum of chemical potential for one element occurs in its elemental phase. Combined with formula (2), *μ*_*O*_ is restricted in following range:


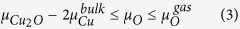


which can be rewritten as:


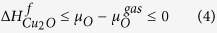


**Figure 2:**
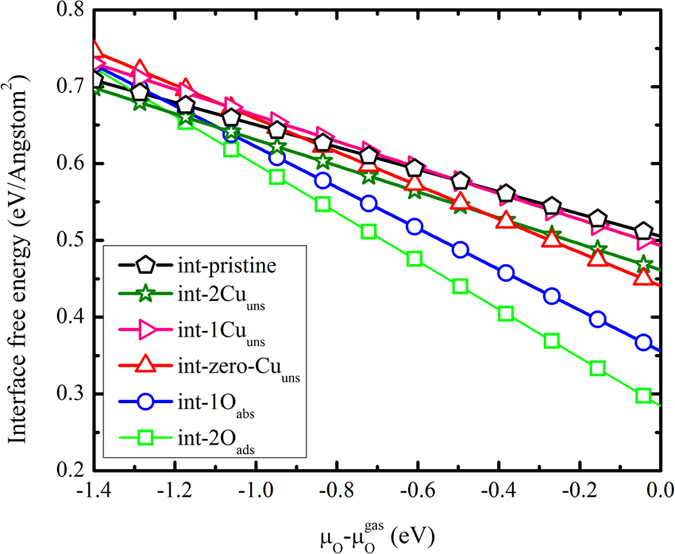
Dependence of interface free (formantion) energy for different structures on the oxygen chemical potential. The int-2O_ads_ structure is found to be the most stable interface structure.

The calculated 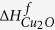 is −1.24 eV (See Supplementary Information Table S1), which is consistent with generalized gradient approximation (GGA) result^29^. The 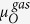, half of an oxygen molecule energy, is −4.92 eV. The interface formation energies of different interfaces are plotted in Fig. 2. All structures tend to become more stable as *μ*_*O*_ increases. The int-2O_ads_ structure possesses the lowest interface energy when 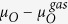 is in the range of −1.2 to 0.0 eV, which agrees with the reference work^29^.

### Microscopic mechanism of the ferromagnetism induced by Cu vacancy

**Figure 3:**
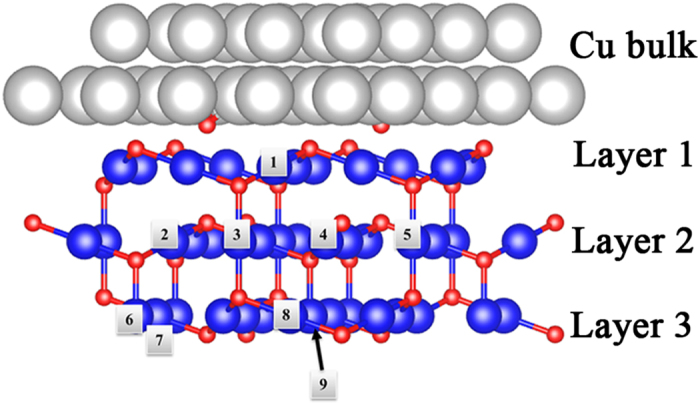
The numbers from 1 to 9 demonstrate the possible V_Cu_ locations in Cu/Cu_2_O int-2Oads interface after introducing one V_Cu_.

Since the Cu vacancies are closely correlated with the interface FM^25^, one V_Cu_ is introduced into in the most stable int-2O_ads_ interface. The possible V_Cu_ locations are labeled as int-2O_ads_-V_Cu_(*n*) in Fig. 3, where *n* denotes the location number. Following the definition of Cu atom in the Cu_2_O surface (see Supplementary Information Figure S1), two types of Cu vacancy (Cu_uns_ and Cu_sat_) are called as “uns” and “sat” in Table 1. Through the collinear spin-polarized calculations, the charge transfer from Cu to Cu_2_O is characterized based on the Bader charge analysis (Table 1)^30^, from which we find that Cu_2_O gains ~2.6 electrons. Such charge transfer originates from the different work functions (WF) between Cu (WF: 4.6 eV) and Cu_2_O (WF: 4.8 eV)^31^,^32^. Among the int-2O_ads_-V_Cu_(*n*) systems, int-2O_ads_-V_Cu_(1) exhibits the largest charge transfer (~2.8 *e*) owing to the new bond formation between the Cu_2_O and the Cu surface.

**Figure 4:**
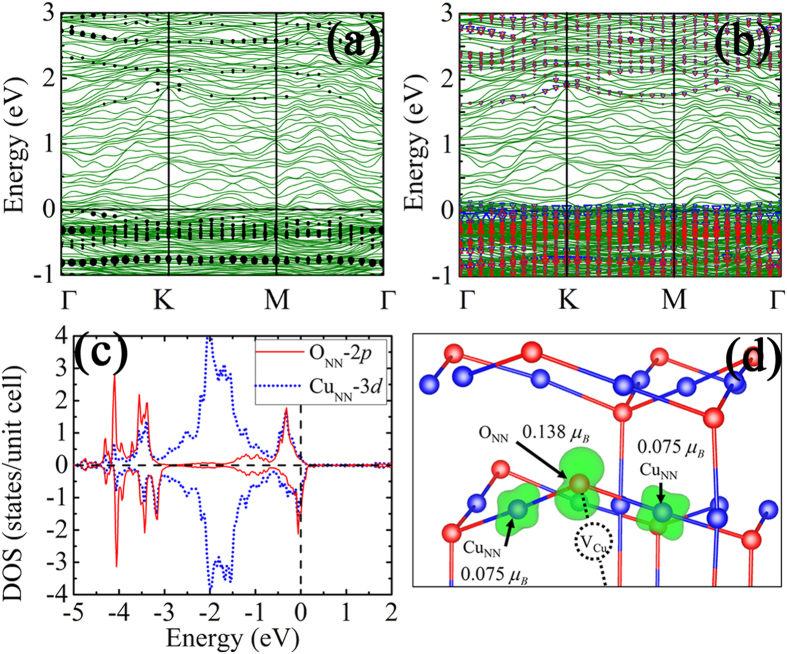
(**a**) The spin-restricted band structure of defect-free int-pristine interface, and the dots from the contribution by Cu_2_O-Layer 2. (**b**) The spin-polarized band structure of int-2O_ads_-V_Cu_(2). The up (down) triangles represent the spin-up (down) states of Cu_2_O-Layer 2. (**c**) The projected DOS of the O_NN_ and Cu_NN_ in int-2O_ads_-V_Cu_(2) interface. (**d**) The spin-densities of the O_NN_ and Cu_NN_ and the corresponding magnetic moments within the atom sphere of radius 0.8 Å.

According to Table 1, it is noticed that not all kinds of V_Cu_ introduce a large magnetic moment into the interface system. Only vacancy at Cu_sat_ in Layer 2 contributes a relatively large magnetic moment (0.4 to 0.5 μ_B_) among all these structures. In order to unveil the FM mechanism, the interface band structures of defect-free int-2O_ads_ and int-2O_ads_-V_Cu_(2) are calculated in Fig. 4(a,b). The green lines represent the total band structure of the interface. The dots and the triangles indicate the contribution from Layer 2 (see Supporting Information Figure S1) where the V_Cu_(2) locates, and the size denotes the weight. Compared with the bulk contribution, more localized surface states are observed near the *E*_F_, and the spin-up and spin-down states are splitted, indicating the Stoner instability. The existence of each V_Cu_ leaves two dangling bonds with respect to its two nearest-neighbor oxygen atoms (O_NN_). These dangling bonds may play a key role to introduce the FM. For more direct visual confirmation, the spin resolved projected density of states (PDOS) of O_NN_ 2*p* and the 3*d* orbitals of the Cu atom (Cu_NN_) bonding with the O_NN_ are plotted in Fig. 4(c). Around the *E*_F_, the O_NN_-2*p* spin-up anti-bonding states (−1 to 0 eV) are mostly occupied and the spin-down anti-bonding states are partially occupied. Meanwhile, due to the anti-bonding states composed by O_NN_-2*p* and Cu_NN_-3*d* orbitals, the Cu_NN_-3*d* orbitals show spin splitting as well. To compare the local magnetic moment within the same range, the spin-density distribution of int-2O_ads_-V_Cu_(2) is visualized within an atom sphere radius 0.8 Å which accords to the Wigner Seitz radius of oxygen atom (Fig. 4(d)). It demonstrates that the upper O_NN_ contributes a relatively large proportion of magnetic moment (0.138 μ_B_), while each Cu_NN_ produces 0.075 μ_B_. Even when the radius is increased to 1.0 Å, the local magnetic moment on O_NN_ and Cu_NN_ are 0.147 μ_B_ and 0.08 μ_B_, respectively, which is quite similar to the above results. In a word, in terms of the FM origin, the magnetic moment at the Cu/Cu_2_O interface is mainly from O_NN_ and the Cu_NN_ atoms. The magnetic moment on Cu_NN_ results from the spin-splitting 3*d* orbitals which forms anti-bonding states with O_NN_-2*p* orbitals.

**Figure 5:**
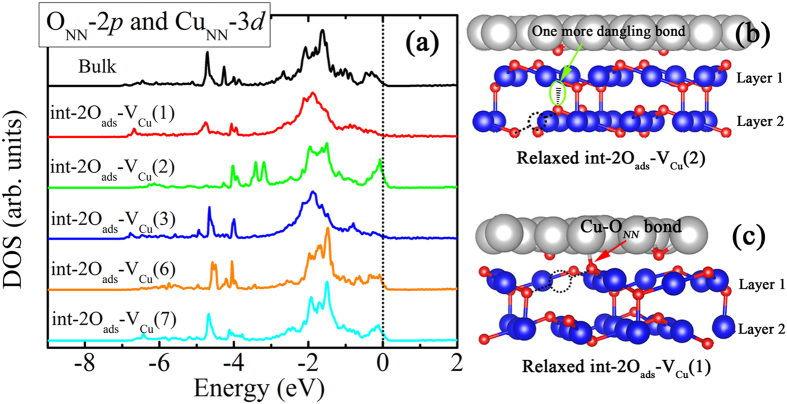
(**a**) The PDOS of O_NN_-2*p* and corresponding Cu_NN_-3*d* orbitals in int-2O_ads_-V_Cu_(n) interface. For clarity, the PDOS for two types of V_Cu_ structures, “uns” and one “sat” in each layer in Fig. 5, and the O_NN_ with the more dangling bonds are presented. (**b**) The relaxed int-2O_ads_-V_Cu_(2) interface structure. Two dangling bonds of O_NN_ are observed due to the natural presence of “uns” type V_Cu_ in Layer 1. (**c**) The relaxed int-2O_ads_-V_Cu_(1) structure. The O_NN_ actually forms a new bond with one Cu atom from upper Cu surface, resulting only one dangling bond of O_NN_. The dotted circles represent the location of V_Cu_.

To further illustrate the origin of the FM contributed by the O_NN_, the PDOS of the O_NN_-2*p* and Cu_NN_-3*d* orbitals in different locations of V_Cu_ are presented in Fig. 5. Referring to Fig. 3, the PDOS for two types of V_Cu_ locations, one “uns” and one “sat” in each layer, are provided here. Each V_Cu_ has two O_NN_ atoms, and for clarity, only the PDOS of O_NN_ with more dangling bonds are shown here. In the bulk Cu_2_O, the O_NN_-2*p* and Cu_NN_-3*d* orbitals generate large localized defect states in the range from −5.0 eV to −4.0 eV below the VBM. Meanwhile, a delocalized band near the VBM is also created. The state around the *E*_F_ indicates that the hole produced by V_Cu_ and occupies a valence band like perturbed-host state (PHS)^33^. Comparing with the bulk Cu_2_O, the O_NN_-2*p* and Cu_NN_-3*d* orbitals also create localized bonding states from −5.0 eV to −4.0 eV below the *E*_F_ and delocalized states around the *E*_F_ when V_Cu_ is not at site 2. However, besides the deep localized states in int-2O_ads_-V_Cu_(2) moving up to the range from −4.0 eV to −3.0 eV, the prominently localized states appear around the *E*_F_. In other words, the Femi level could be pinned by these localized states. To understand this pinning phenomenon, we calculate the energy difference (∆*E*) between the *E*_F_ and the VBM of Cu_2_O. For the defect-free int-2O_ads_ interface, this energy difference is 0.12 eV. However in the int-2O_ads_-V_Cu_(2) interface, ∆*E* is also ~0.12 eV. Since the localized defect states induced by the V_Cu_(2) would pin the *E*_F_ at the certain location and the charge transfer would not obviously shift *E*_F_, the FM occurs consequently.

To investigate why only certain V_Cu_ could create localized states around the *E*_F_, we focus on the dangling bonds character of the O_NN_. Generally, the O atoms in Cu_2_O bulk form four bonds with the nearest four Cu atoms. Hence one O_NN_ around the V_Cu_ forms bonds with three other Cu atoms, generating one dangling bond. The spin-polarized calculation for a 2 × 2 × 2 bulk Cu_2_O supercell with one Cu vacancy gives the magnetic moment per atom less than 0.0001 μ_B_, indicating a non-magnetic state. However, in the int-2O_ads_-V_Cu_(2) interface, the upper O_NN_ is merely bonding with two Cu atoms due to the natural “uns”-V_Cu_ presence in Layer 1 (Fig. 5(b)). Therefore, the unique int-2O_ads_-V_Cu_(2) interface structure provides O_NN_ one more dangling bonds, which leads to the weaker *p-d* hybridization and a more localized O-2*p* wave functions. For int-2O_ads_-V_Cu_(1), a new bond between the O_NN_ and the Cu atom in above Cu(111) surface in the relaxed structure, as shown in Fig. 5(c). The new bond length is 1.95 Å, which is shorter than that in other int-2O_ads_-V_Cu_(n) structures (~2.07 Å). Thus, the new bond formation leads to the delocalized O_NN_-2*p* orbitals and the quenching of spin magnetic moment. It implies that once the dangling bonds are compensated, the larger magnetic moment shrinks.

### Modulation of the ferromagnetism driven by interfacial Cu diffusion

**Figure 6:**
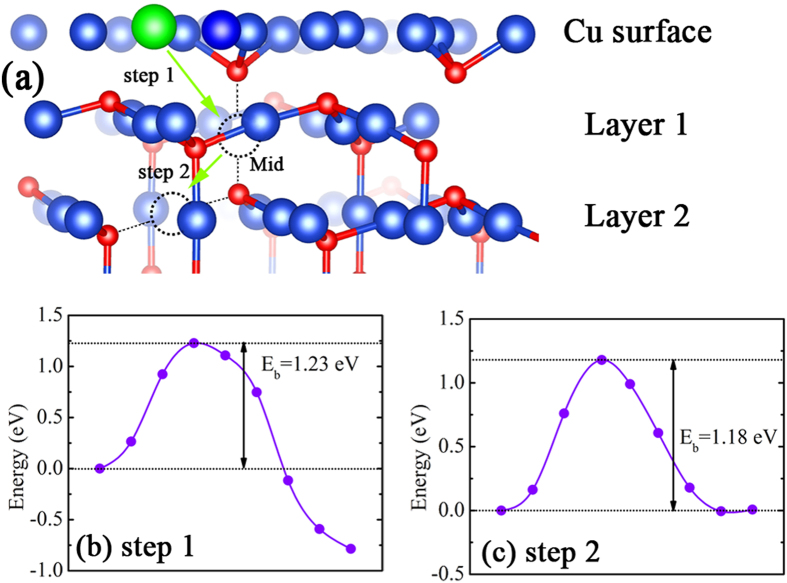
(**a**) Cu diffusion route from Cu(111) surface to the V_Cu_ site in int-2O_ads_-V_Cu_(2) structure. The energy curves in (**b**,**c**) corresponding to the first and the second step during the diffusion. The dotted circles represent the different reaction coordinates in NEB calculations.

The FM in Cu/Cu_2_O interface is sensitive to the annealing process, in which the amount of the V_Cu_ responsible for the FM could be tunable. It is important to investigate the feasibility of the Cu diffusion through the interface. Actually, the Cu diffusion from Cu into Cu_2_O is observed in previous experiments^34^,^35^. Such diffusion may degrade the electrical performance in Cu_2_O thin-film transistors^36^ and modulate the magnetism during the Cu oxidation. Up to date, the theoretical Cu diffusion process and the energy barrier (*E*_*b*_) in Cu/Cu_2_O contact are not well understood. Hence, a thoroughly study on the energy barrier related to the Cu diffusion and its influence on the FM is performed by climb image nudge elastic band (CI-NEB) calculations. As the int-2O_ads_-V_Cu_(2) structure has the largest magnetic moment, we focus on the Cu diffusion in this structure. As the V_Cu_ in int-2O_ads_-V_Cu_(2) structure is located in Layer 2 (see Fig. 3), the diffusion process could be divided into two steps as shown in Fig. 6(a). The first step is one Cu atom moving from the Cu(111) surface into the natural V_Cu_ located in Layer 1, leaving a vacancy on Cu(111) surface behind. Following the first step, this specific Cu atom further diffuses into V_Cu_(2) in Layer 2 (see Fig. 3). Once the V_Cu_ in Layer 2 is compensated, the FM almost vanishes. Within the CI-NEB calculations, the energy barrier (*E*_*b*_) is found to be 1.23 eV in the first step, and the total energy drops by 0.79 eV in the final stage, as plotted in Fig. 6(b). Such energy barrier is ~0.2 eV higher than the activation energy (*E*_*a*_) in the Cu_2_O growth by oxidation (~1.0 eV)^37^,^38^. The Cu_2_O growth from Cu oxidation is closely related with the Cu diffusion from Cu to Cu_2_O^39^:


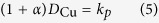


where *α* and *D*_Cu_ are the degree of ionization of defects and diffusion coefficient of Cu, respectively. *k*_*p*_ denotes the parabolic rate which could be obtained by:


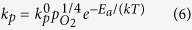


where 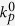 is a prefactor and 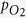 represents the partial pressure in oxidation. Actually, the activation energy (*E*_*a*_) is the energy barrier (*E*_*b*_) in the Cu oxidation process, which determines the Cu diffusion. Please refer to the link http://en.wikipedia.org/wiki/Activation_energy for the definition of activation energy. According to the Arrhenius formula, the relationship between *D*_Cu_ and the diffusion energy barrier (*E*_*b*_) can be written in the following equation:


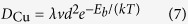


Thus,


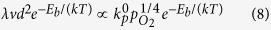


in which *λ* is a dimensionless factor, *ν* and *d* indicate the vibration frequency (normally 10^12^ ~ 10^13^ s^−1^) and the jump distance, respectively. When 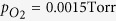 (0.2 Pa)^25^, 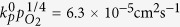 (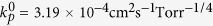 in ref. 40), *d* should be several angstroms to make *D*_Cu_ compatible for both sides of Eq. (8), which demonstrates that the Cu diffusion (~4 Å in the first step and ~3 Å in the second step) could be achievable. In Fig. 6(c), the *E*_*b*_ in the second step is 1.18 eV which is slightly less than that in the first step. The lower *E*_*b*_ in the second step implies that the Cu further moves easily to the V_Cu_ in the second layer of Cu_2_O(111) as well once it reaches the Cu_2_O surface.

At the middle location (labeled as “Mid” in Fig. 6) in the whole diffusion process, the spin-polarized calculation is performed. The total magnetic moment, only 0.0003 μ_B_, indicates that the diffused Cu suppresses the FM. At the “Mid” site, the diffused Cu forms bonds with the O_NN_ and the local magnetic moment drops significantly because partial dangling bonds of O_NN_ are compensated. A higher *D*(*E*_F_) of the Stoner criterion is no longer satisfied, which leads to the quenching of the FM. Thus during the growth of Cu_2_O under Cu oxidation, the annealing treatment would influence the Cu diffusion. To further quantify the diffusion feasibility, the approximated diffusion time can be solved by^39^:


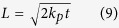


where *L* denotes the obtained oxidized layer (Cu_2_O) thickness after the oxidization duration of *t*. When *E*_*b*_ = 1.2 eV and oxygen partial pressure is 0.2 Pa, the first step diffusion (~4 Å) would be finished in *about 40* *minutes*. This result explains the experimental observation^25^. Therefore, the FM modulated by the annealing process in experiment is actually realized by controlling the amount of interfacial V_Cu_ through Cu diffusion within the Cu/Cu_2_O interface.

## Conclusion

To summarize, the FM in Cu/Cu_2_O contact is induced by V_Cu_ around the Cu/Cu_2_O interface. Only the interface structure with the “sat” type V_Cu_ in the second layer possesses a relatively large magnetic moment due to two dangling bonds of O_NN_. The *E*_F_ is pinned in the O_NN_-2*p* and Cu_NN_-3*d* localized states and a large *D*(*E*_F_) is achieved by the charge transfer from Cu to Cu_2_O. Once the V_Cu_ is compensated by the diffused Cu atom, the number of dangling bonds reduces and the FM vanishes. A moderate energy barrier (~1.2 eV) guarantees the feasibility to modulate the FM by controlling Cu diffusion in experiment. These results offer a comprehensive understanding about the microscopic mechanism of the FM and its modulation by V_Cu_ in Cu/Cu_2_O interface. Also, our calculations provide an insight to understand and tune the FM relevant with defects in other metal/oxides contacts.

## Calculation methods

All the calculations are performed using Vienna *ab initio* simulation package (VASP). The generalized gradient approximation (GGA) with exchange-correlation function of Perdew-Burke -Ernzerhof (PBE) is chosen^40^. The energy cutoff of 400 eV is selected and the electronic optimization stops when the total energies of neighboring optimization loops differ below 10^−5^ eV in all the calculations. A 7 × 7 × 7 Monkhorst-Pack *k*-point mesh is set up in the bulk calculations. To avoid the interaction between periodic images, the vacuum thickness is set up to 15 Å for the surface and interface slab structures. The Γ-centered 5 × 5 × 1 *k*-point mesh is adopted in slab calculations. For the structural relaxation, the force on each atom is chosen to be less than 0.001 eV/Å in bulk calculations and less than 0.05 eV/Å in surface and interface calculations. The Cu diffusion paths are calculated by the climb image nudge elastic band (CI-NEB) method^41^.

## Additional Information

**How to cite this article**: Li, H.-B. *et al.* Electronic Structure and Ferromagnetism Modulation in Cu/Cu_2_O Interface: Impact of Interfacial Cu Vacancy and Its Diffusion. *Sci. Rep.* **5**, 15191; doi: 10.1038/srep15191 (2015).

## Supplementary Material

Supplementary Information

## Figures and Tables

**Table 1:** Total magnetic moment and the amount of charge transfer from Cu to Cu_2_O in int-2O_ads_ with one V_Cu_ at different locations.

Structure	Type of V_Cu_	V_Cu_ location	Charge transfer from Cu to Cu_2_O (*e*)	Total magnetic moment (μ_B_)
int-2O_ads_	N/A	N/A	2.58	0.000
int-2O_ads_-V_Cu_(1)	sat	Layer 1	2.79	0.000
int-2O_ads_-V_Cu_(2)	sat	Layer 2	2.57	0.501
int-2O_ads_-V_Cu_(3)	uns		2.60	0.000
int-2O_ads_-V_Cu_(4)	sat		2.57	0.420
int-2O_ads_-V_Cu_(5)	uns		2.62	0.000
int-2O_ads_-V_Cu_(6)	uns	Layer 3	2.58	0.002
int-2O_ads_-V_Cu_(7)	sat		2.58	0.000
int-2O_ads_-V_Cu_(8)	sat		2.58	0.001
int-2O_ads_-V_Cu_(9)	uns		2.58	0.000
