# Supplementary material for: Electronic Structure and Ferromagnetism Modulation in Cu/Cu2O Interface: Impact of Interfacial Cu Vacancy and Its Diffusion
Source: Sci Rep. 2015 Oct 19;5:15191. doi: 10.1038/srep15191 (PMC4609911; doi:10.1038/srep15191)
Supplement: Supplementary Information [file srep15191-s1.pdf]

Electronic Structure and Ferromagnetism Modulation in Cu/Cu_2O Interface: Impact of Interfacial Cu Vacancy and Its Diffusion

Hao-Bo Li[1], Weichao Wang[1,4], Xinjian Xie[2], Yahui Cheng[1], Zhaofu Zhang[1], Hong Dong[1], Rongkun Zheng[3], Wei-Hua Wang*[,1], Feng Lu*[,1], Hui Liu[1]

[1]*Department of Electronics and Tianjin Key Laboratory of Photo-Electronic Thin Film Device and Technology, Nankai University, Tianjin, 300071, China*

[2]*School of Materials Science and Engineering, Hebei University of Technology, Tianjin, 300130, China*

[3]*Tianjin School of Physics, the University of Sydney, NSW, 2006, Australia*

[4]*Department of Materials Science and Engineering, The University of Texas at Dallas, Richardson, Texas 75080, United States*

Bulk and surface calculations of Cu and Cu_2O

For comparison, the bulk and the clean (111) surfaces of Cu and Cu_2O are studied before investigating the Cu/Cu_2O interface. The calculated lattice constant of Cu bulk is 3.63 Å, which is in well agreement with the experimental result of 3.61 Å[1]. For the clean surface of Cu(111), a five-layer slab model with a vaccum space of 15 Å is adopted. The interlayer relaxation values $\Delta_{ij} = (d_{ij} - d)/d$, where *i* and *j* denote the different layers and *d*=2.10 Å is the bulk interlayer spacing. The first and second relaxation values are Δ_{12}

= −0.9% and Δ_{23} = −0.3%, which well accord with experimental results of Δ_{12} = −0.7%, Δ_{23} = −0.3% and other DFT calculations[2,3].

Cu_2O is one of the most common Cu oxides with a cuprite structure, and a unit cell contains six atoms (Cu_4O_2) as shown in Figure S1(a). In Table S1, the bond length, enthalpy of formation ($\Delta H^{f}_{Cu_2O}$) and the band gap (E_g) are listed. The optimized lattice constant, 4.31 Å, is in line with theoretical and experimental results[4]. Similar to the previously theoretical work[5], the obtained band gap is as small as 0.61 eV due to the well-known problem of DFT, which underestimates band gaps of semiconductors.

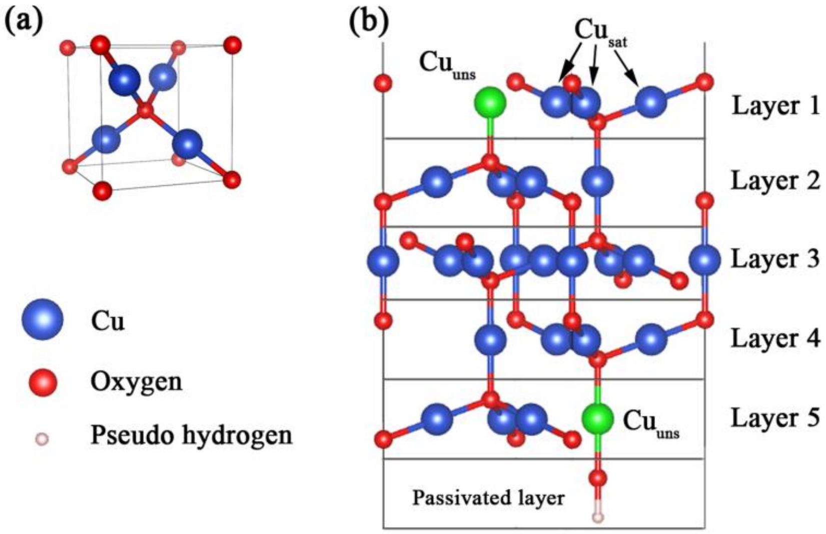

Figure S1. (a) Cu_2O bulk structure. (b) Five layers $Cu_2O(111)$ surface slab model. The Cu_{uns} in bottom layer is fixed and is passivated by oxygen and 1.5 *e* pseudo-hydrogen atoms.

Table S1. Optimized lattice constant, bond length, formation entropy and band gap of bulk Cu_2O.

	a_0 (Å)	$d_{Cu\text{-}O}$ (Å)	$d_{Cu\text{-}Cu}$ (Å)	$\Delta H^{f}_{Cu_2O}$ (eV)	Eg (eV)
Present work (GGA+PBE)	4.31	1.87	3.04	-1.24	0.61
Dmol (GGA+PBE)[5]	4.32	1.87	3.05	-1.24	0.64
Experiment[4]	4.27	1.84	3.02	-1.75	2.17
PWPP[3]	4.32	1.88	3.07		
FPLAPW[6]	4.30	1.86	3.04		0.5

The Cu_2O(111) 2×2 surface is cleaved as illustrated in Figure S1(b), containing five sandwich-like O-Cu-O layers. Both chemical saturated (Cu_{sat}) and unsaturated (Cu_{uns}) Cu atoms are labeled in Layer 1. Cu_{sat} forms bonds with O atoms within the same layer, while the Cu_{uns} bonds with O atoms in the adjacent layers. In order to mimic the Cu_2O bulk phase, the bottom layer (Layer 5) is fixed during the structural relaxations and the Cu_{uns} atoms are passivated by oxygen and pseudo-hydrogen atoms with 1.5 *e*. According to the density of the states (DOS) in Figure S2, the surface states are observed in the band gap[3]. From the local density states analysis (right in Figure S2), the surface states are mostly contributed by Layer 1 and 2. Meanwhile, the band structure (left in Figure S2) indicates that Layer 3 and Layer 4 (black dots) preserve the Cu_2O bulk properties. The above results ensure that five layers slab model is suitable for the further interface exploration.

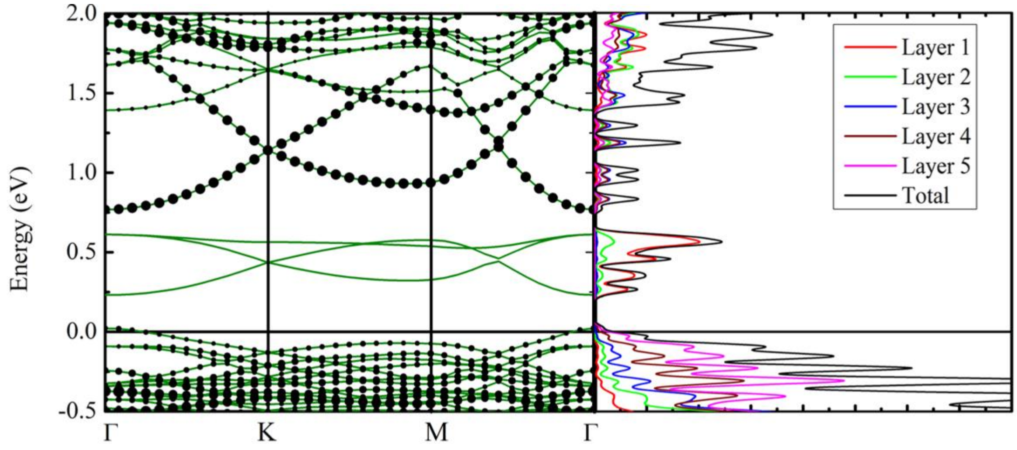

Figure S2. Band structure and DOS of the pristine $Cu_2O(111)$ surface. The black dots denote the bulk-like contributions by Layer 3 and Layer 4.

Optimization of Cu/Cu_2O interface structure

In order to obtain a reasonable interface, prisitine Cu_2O surface is moved along x, y and z directions on the Cu surface to determine the location with the lowest interface energy. The movement distance and the corresponding total energy of the interface are exhibited in Figure S3. The interface top view with the lowest energy is as shown in Figure S3(d), which is adopted as the initial pristine Cu/Cu_2O structure in further calculations.

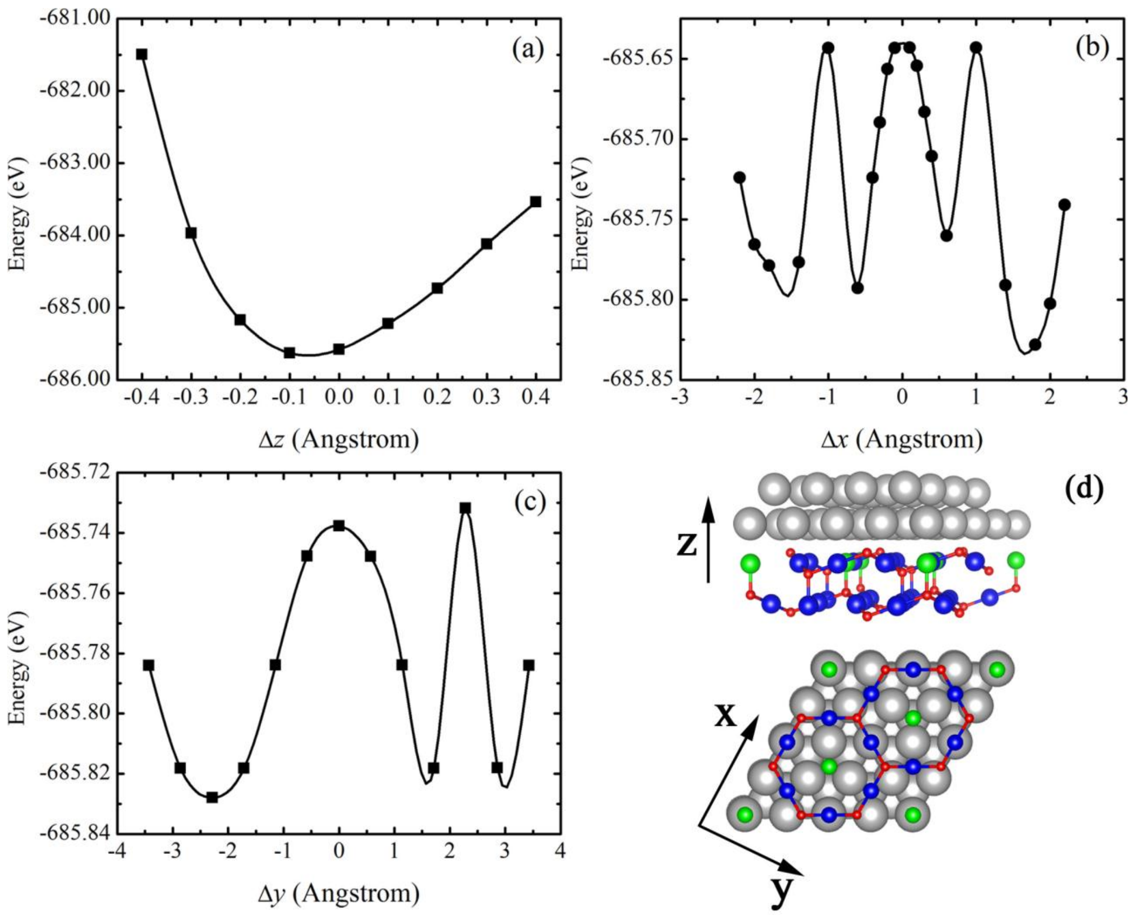

Figure S3. (a-c) Total energy dependence on movement of Cu_2O along *x*, *y* and *z* axis. (d) *x*, *y*, *z* axis and the most energetic favorable pristine Cu/Cu_2O interface structure.

References

1. Kittel, C. *Introduction to Solid State Physics*, Wiley, New York, (1996).

2. Lindgren, S. Å., Walldén, L., Rundgrén, J. & Westrin, P. Low-energy electron diffraction from Cu(111): Subthreshold effect and energy-dependent inner potential; surface relaxation and metric distances between spectra. *Phys. Rev. B* **29,** 576-588 (1984).

3. Soon, A., Söhnel, T. & Idriss, H. Plane-wave pseudopotential density functional theory periodic slab calculations of CO adsorption on $Cu_2O(111)$ surface. *Surf. Sci.* **579,** 131-140 (2005).

4. Lide, D. R. *CRC Handbook of Chemistry and Physics*, *Internet version 2005*, CRC Press, Boca Raton, FL, (2005).

5. Soon, A., Todorova, M., Delley, B. & Stampfl, C. Oxygen adsorption and stability of surface oxides on Cu(111): A first-principles investigation. *Phys. Rev. B* **73,** 165424 (2006).

6. Martínez-Ruiz, A., Moreno, M. G. & Takeuchi, N. First principles calculations of the electronic properties of bulk Cu_2O, clean and doped with Ag, Ni, and Zn. *Solid State Sci.* **5,** 291-295 (2003).
